# Supplementary material for: Patterns and controlling factors of soil carbon sequestration in nitrogen-limited and -rich forests in China—a meta-analysis
Source: PeerJ. 2023 Jan 18;11:e14694. doi: 10.7717/peerj.14694 (PMC9864202; doi:10.7717/peerj.14694)
Supplement: Table S1 — The citations, amount of N addition, N form, ecosystem type, mean annual temperature (MAT), mean annual precipitation (MAP), and location for all observations used in the meta-analysis. [file peerj-11-14694-s001.doc]

# Table S1

The citations, amount of N addition, N form, ecosystem type, mean annual temperature (MAT), mean annual precipitation (MAP), and location for all observations used in the meta-analysis.

| **N°** | **Citation** | **N deposition  (kg N ha^-1^ yr^-1^)** | **N form** | **Forest Type** | **MAT** | **MAP** | **Location** |
| --- | --- | --- | --- | --- | --- | --- | --- |
| 1 | (Deng et al., 2010) | 29.5 | NH_4_NO_3_ | Subtropical | 21.5 | 1750 | 23°20'N, 113°30'E |
| 2 | (Fu et al., 2020) | 18.5 | NH_4_NO_3_ | Subtropical | 15.7 | 995.4 | 31°48'-31°58N, 117°11-117°22'E |
| 3 | (Shi et al., 2019) | 8.5 | NH_4_NO_3_ | Boreal | -5.4 | 550 | 51°00′N, 122°03′E |
| 4 | (Mo et al., 2007) | 37 | NH_4_NO_3_ | Tropical |  | 1927 | 112°100'E, 23°100'N |
| 5 | (Yan et al., 2016) | 13.33 | NH_4_NO_3_ | Temperate | 12.3 | 728 | 48°02′–48°12′ N, 128°58′–129°15′ E |
| 6 | (Liu et al., 2017) | 36.3 | NH_4_NO_3_ | Subtropical | 19.1 | 1750 | 26°19′55′′ N, 117° 36′53′′ E |
| 7 | (Gao et al., 2014) | 22.6 | NH_4_NO_3_ | Subtropical |  |  | 29°52'N, 121°39'E |
| 8 | (Song et al., 2017) | 12.93 | NH_4_NO_3_ | Temperate | −0.3 | 805 | 47° 10′ 50″ N, 128° 53′ 20″ E |
| 9 | (Tu et al., 2011) | 25.5 | NH_4_NO_3_ | Subtropical |  |  | 29°95′ N, 103°38′ E |
| 10 | (Lu et al., 2019) | 34 | NH_4_NO_3_ | Subtropical | 26.4 | 1900 | 24°22'~24°31'N, 113°05'~113°31'E |
| 11 | (Yu et al., 2020) | 40 | NH_4_NO_3_ | Subtropical | 1700 | 21.4 | 23°09′-23°12′N, 112°31′-112°34′E |
| 12 | (Li et al., 2019b) | 19 | NH_4_NO_3_ | Temperate | 645.3 | 4.7 | 43°41′30″N-43°49′N, 125°26′15″E-125°33′45″E |
| 13 | (Ma et al., 2021) | 9 | NH_4_NO_3_ | Tropical | 19.7 | 2198 | 18°43'N, 108°53'E |
|  | / | 16 | NH_4_NO_3_ | Subtropical | 19.7 | 2198 | 27°39'N, 117°57'E |
|  | / | 15 | NH_4_NO_3_ | Temperate | 19.7 | 2198 | 39°58'N, 115°26'E |
|  | / | 7 | NH_4_NO_3_ | Boreal | 19.7 | 2198 | 50°56'N, 121°30'E |
| 14 | (Peng et al., 2017) | 10 | NH_4_NO_3_ | Subtropical | 10 | 1395 | 29°32′35″N, 103°15′41″E |
| 15 | (Ma et al., 2018) | 14.98 | NH_4_NO_3_ | Subtropical | 8.7 | 625 | 36°40N–36°47′N, 111°59′E–112°05′E |
| 16 | (Li et al., 2019a) | 22.6 | Urea | Subtropical | 0.13 | 414.98 | 37°11′N, 102°46′E |
| 17 | (Zhang et al., 2017) | 33.5 | NH_4_NO_3_ | Subtropical | 15.6 | 1420 | 30°01′N, 117°21′E |
| 18 | (Lu et al., 2012) | 29.5 | NH_4_NO_3_ | Subtropical | 21 | 1927 | 30°01′N, 117°21′E |
| 19 | (Chen et al., 2012) | 14.98 | NH_4_NO_3_ | Subtropical | 21.5 | 1956 | 23°10′N, 112°10′E |
| 20 | (Liu et al., 2020) | 25 | NH_4_NO_3_ | Boreal | -2.4 | 500 | 51°05′-51°39′ N, 125°07′-125°50′ E |
| 21 | (Chen et al., 2017) | 23 | NH_4_NO_3_ | Temperate | 2.7 | 871.6 | 42.517 N, 127.783 E |
| 22 | (Chen et al., 2018) | 39 | NH_4_NO_3_ | Subtropical | 6.6 | 1490 | 29'42°N, 103'14°E |
| 23 | (Chen et al., 2019) | 23 | NH_4_NO_3_ | Temperate | 3.6 | 740 | 41°42'N, 127°38'E |
| 24 | (Chen et al., 2020) | 21.95 | NH_4_NO_3_ | Subtropical | 17.5 | 1900 | 27°42'N, 117°45'E |
| 25 | (Cheng et al., 2018) | 20 | Urea | Temperate | 3.6 | 700 | 41°42'N, 127°38'E |
| 26 | (Wang et al., 2016) | / | Urea | Temperate | 9.9 | 548 | 36°18′N, 111°45′E |
| 27 | (Wang et al., 2019) | 117 10 | NH_4_NO_3_ | Subtropical | 16 | 1626 | 30°01'47” N, 117°21'23” |
| 28 | (Du et al., 2014) | 20 | Urea | Temperate | −1.4 | 450.1 | 42°10′–42°50′N, 117°12′–117°30′E |
| 29 | (Lu et al., 2010) | 31 | NH_4_NO_3_ | Tropical | 21 | 1927 | 23°10'N, 112°10'E |
| 30 | (Wang et al., 2009) | 25 | NH_4_NO_3_ | Subtropical | 20.9 | 1927 | 23°08′N, 112°35′E |
| 31 | (Fang et al., 2007) | 35.6 | NH_4_NO_3_ | Subtropical | 21 | 1927 | 23°19′N, 112°19′E |
| 32 | (Duan et al., 2019) | 12.82 | NH_4_NO_3_ | Tropical | 15.6 | 1420 | 22°06'N, 106°43'E |
| 33 | (Zhang et al., 2019a) | 7.91 | NH_4_NO_3_ | Temperate | 11 | 600 | 36°040 N, 112°060 E |
| 34 | (Zhou et al., 2019) | 40 | NH_4_NO_3_ | Subtropical | 18.7 | 2025 | 26°110'N, 117°280'E |
| 35 | (Fang et al., 2007) | 35.6 | NH_4_NO_3_ | Tropical | 21 | 1,927 | 23°19′N, 112°19′E |
| 36 | (Xiao et al., 2020) | 43 | NH_4_NO_3_ | Subtropical | 16.7 | 1469 | 29°16'–29°17'N, 115°42'–15°43'E |
| 37 | (Lu et al., 2013) | 33 | NH_4_NO_3_ | Subtropical | 21 | 1927 | 23°10'N, 112°10'E |
| 38 | (Zhu et al., 2021) | 5.33 | Urea | Temperate | 2 | 500 | 43.47°N, 87.18°E |
| 39 | (Liang et al., 2019) | 2 | NH_4_NO_3_ | Subtropical | 15.2 | 1120 | 36° 52′N, 114° 05′E |
| 40 | (Wang et al., 2017) | 12.82 | NH_4_NO_3_ | Subtropical | 16.5 | 1200 | 26◦40 N, 109◦26 E |
| 41 | (Wende et al., 2020) | 39.2 | NH_4_NO_3_ | Subtropical | 17.2 | 1422 | 28°06′07''N, 113°02'01''E |
| 42 | (WANG et al., 2015) | 12.82 | NH_4_NO_3_ | Subtropical | 16.5 | 1200 | 26°40'–27°09'N, 109°26'–110°08'E |
| 43 | (Zeng et al., 2018) | 7 | Urea | Temperate | -1.4 | 450.1 | 42°10′–42°50′N, 117°12′–117°30′E |
| 44 | (Sun et al., 2014) | 32 | NH_4_NO_3_ | Tropical | -1.4 | 450 | 42°25'N, 117°15'E |
| 45 | (Mo et al., 2008) | 32 | NH_4_NO_3_ | Tropical |  | 1927 | 23°10'N, 112°10'E |
| 46 | (Zhao et al., 2018) | 21.2 | Urea | Temperate | 9.9 | 662 | 36°31’N-36°43’ N, 112°01′E-112°15′E |
| 47 | (Lu et al., 2013) | 33 | NH_4_NO_3_ | Tropical | 21 |  | 23°10'N, 112°10'E |
| 48 | (Yan et al., 2017) | 2.5 | NH_4_NO_3_ | Temperate | 13.6 | 489 | 51°05'–51°39'N, 125°07'–125°50'E |
| 49 | (Zhang et al., 2019b) | 18 | NH_4_NO_3_ | Temperate |  |  | 23°9′41″N, 112°32′36″E |
| 50 | (Tian et al., 2017) | 5.5 | NH4NO3 |  | -5.4 | 481 | 50°56′N, 121°30′E |
|  | / | 7 | NH4NO3 |  | -0.5 | 654 | 48°07′N, 129°11′E |
|  | / | 10.6 | NH4NO3 |  | 9.2 | 1650 | 30°01′N, 117°21′E |
|  | / | 16 | NH4NO3 |  | 18 | 1889 | 27°39′N, 117°57′E |
| 51 | (Zhou et al., 2018) | 13 | NH_4_NO_3_ | Subtropical | 16.2 | 1700 | 30˚03’N, 102˚59’E |
| 52 | (Fan et al., 2014) | 4.2 | Urea | Subtropical |  |  | 26°30′N, 117°43′E |
| 53 | (Lu et al., 2021) | 34.4 | NH_4_NO_3_ | Tropical | 21.9 | 1748 | 23°10'N, 112°10'E |
| 54 | (Zhu et al., 2016) | 43.1 | NH_4_NO_3_ | Tropical | 22.5 | 1543 | 22°34'N, 112°50'E |
| 55 | (Yan et al., 2018) | 2.5 | NH_4_NO_3_ | Boreal | −2.4 | 489.2 | 51°05′–51°39′N, 125°07′–125°50′E |
| 56 | (Zeng and Wang, 2015) | 1.3 | Urea | Temperate | 1.4 | 450.1 | 42°10'–42°50'N, 117°12'–117°30'E |
| 57 | (Wang et al., 2018) | 32 | NH_4_NO_3_ | Tropical | 21 | 1927 | 23°10′N, 112°10′E |
| 58 | (Liu et al., 2013) | 33 | NH_4_NO_3_ | Tropical | 21 |  | 23°109'N, 112°10'E |
| 59 | (Tian et al., 2019) | 73 | NH_4_NO_3_ | Tropical | 21 | 1930 | 23°10′N, 112°10′E |
| 60 | (Song et al., 2020) | 13 | Urea | Temperate | − 1.4 | 450 | 42° 24.723′ N, 117° 14.844′ E |
| 61 | (Jia et al., 2010) | 14.3 | NH_4_NO_3_ | Temperate | 2.8 | 723 | 45°21′-45°25′ N, 127°30′-127°34′E |
|  |  |  |  |  |  |  |  |
