# Supplementary figures and images for: Patterns and controlling factors of soil carbon sequestration in nitrogen-limited and -rich forests in China—a meta-analysis
Source: PeerJ. 2023 Jan 18;11:e14694. doi: 10.7717/peerj.14694 (PMC9864202; doi:10.7717/peerj.14694)

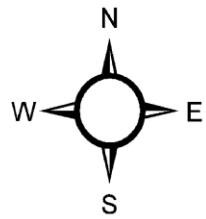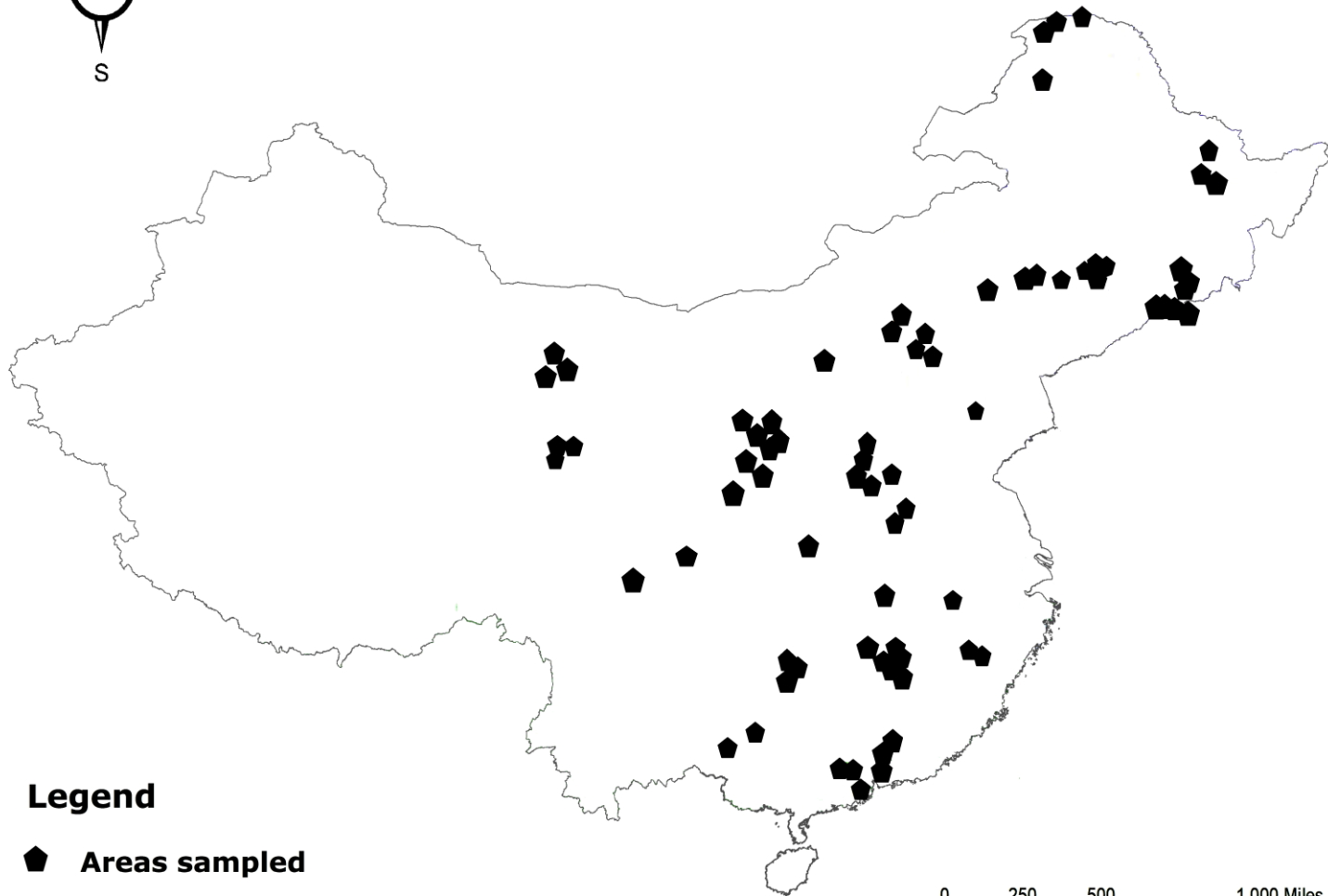

0 250 500 1,000 Miles

1

2

Supplement: Figure S1 [file peerj-11-14694-s003.pdf]

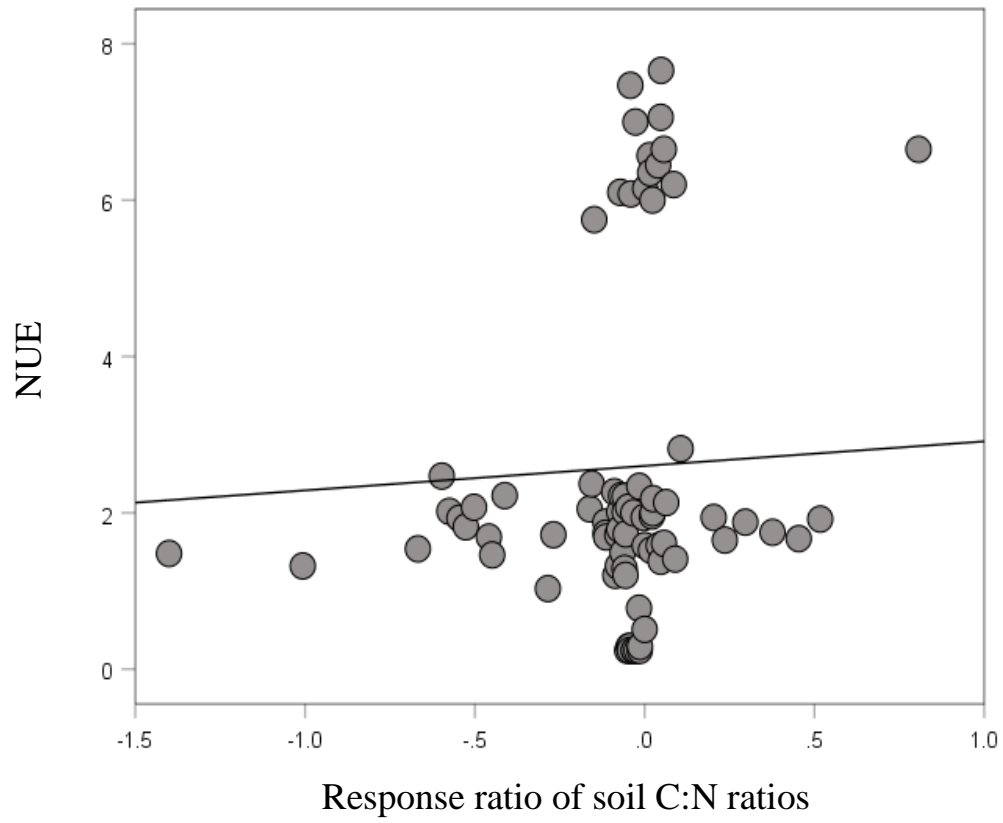

Supplement: Figure S2 [file peerj-11-14694-s004.pdf]

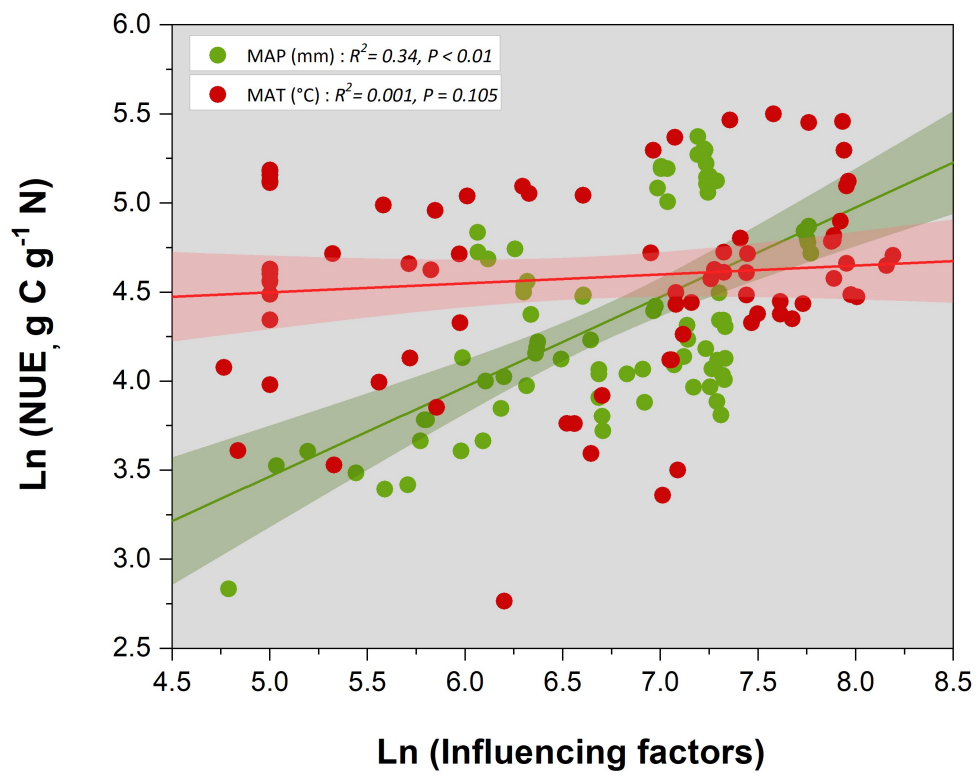

51

52

53

54

Supplement: Figure S3 [file peerj-11-14694-s005.pdf]

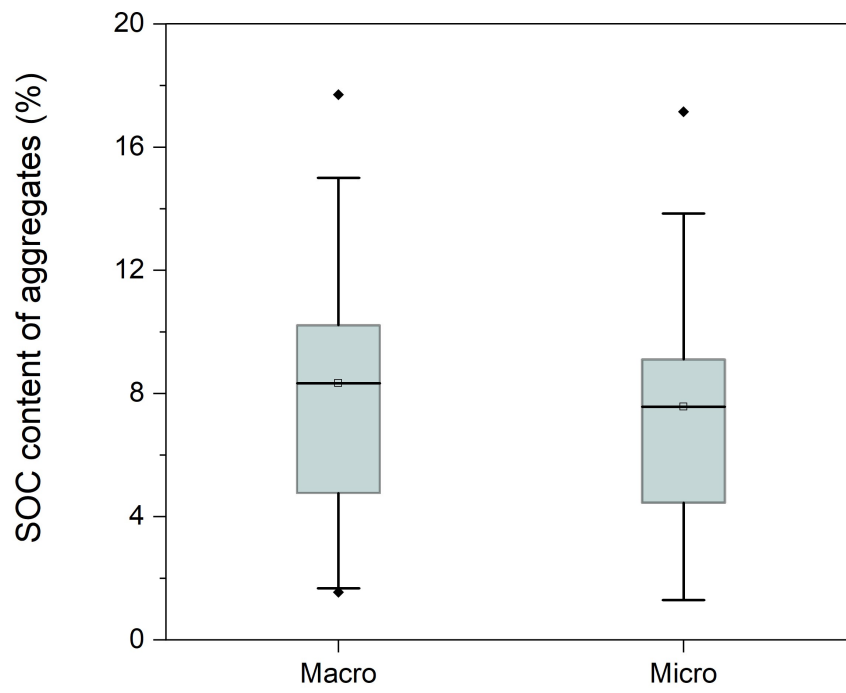

55

56

57

58

Supplement: Figure S4 [file peerj-11-14694-s006.pdf]

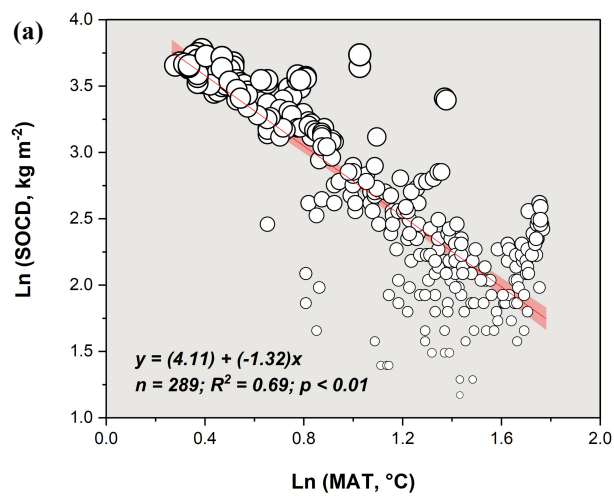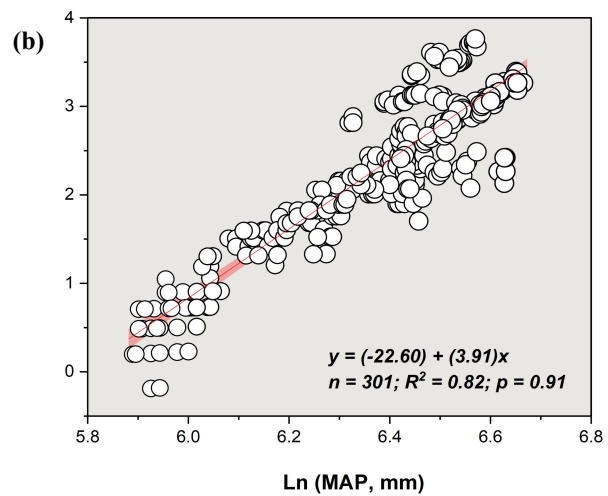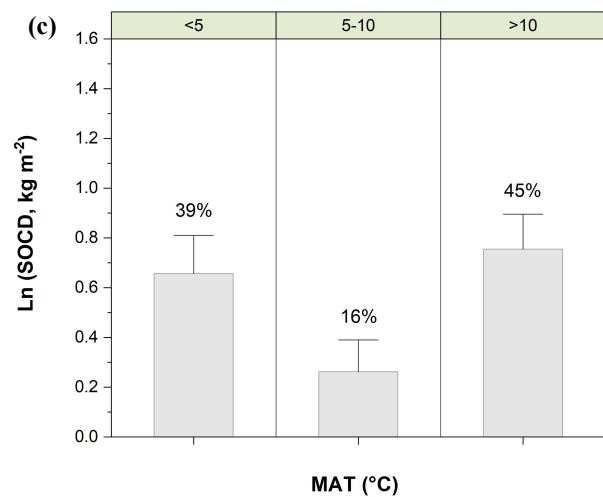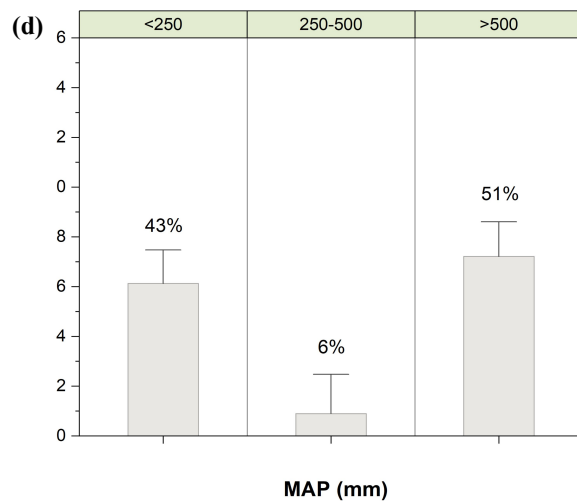

Supplement: Figure S5 — Mean annual temperature (MAT); mean annual precipitation (MAP). The red area around the regression line represents the 95% confidence interval, whereas N is the number of observations. [file peerj-11-14694-s007.pdf]
