# Supplementary material for: Patterns and controlling factors of soil carbon sequestration in nitrogen-limited and -rich forests in China—a meta-analysis
Source: PeerJ. 2023 Jan 18;11:e14694. doi: 10.7717/peerj.14694 (PMC9864202; doi:10.7717/peerj.14694)
Supplement: Supplemental Information 8 [file peerj-11-14694-s008.docx]

| **Section and Topic** | **Item #** | **Checklist item** | **Location where item is reported** |
| --- | --- | --- | --- |
| **TITLE** | | |  |
| Title | 1 | Patterns and controlling factors of soil carbon sequestration in nitrogen-limited and -rich forests in China. A meta-analysis | Manuscript |
| **ABSTRACT** | | |  |
| Abstract | 2 | Soil organic carbon (SOC) management has the potential to contribute to climate change mitigation by reducing atmospheric carbon dioxide (CO2). Understanding the changes in forest nitrogen (N) deposition rates has important implications for C sequestration. We explored the effects of N enrichment on soil carbon sequestration in nitrogen-limited and nitrogen-rich Chinese forests and their controlling factors. Our findings reveal that N inputs enhanced net soil C sequestration by 5.52 to 18.46 kg C kg-1 N, with greater impacts in temperate forests (8.37 to 13.68 kg C kg-1 N), the use of NH4NO3 fertilizer (7.78 kg C kg-1 N), at low N levels (<30 kg C kg-1 N; 9.14 kg C kg-1 N), and in a short period (<3 years; 12.95 kg C kg-1 N). The NUE varied between 0.24 and 13.3 (kg C kg-1 N) depending on the forest type and was significantly controlled by rainfall, fertilizer, and carbon-nitrogen ratio rates. Besides, N enrichment increased SOC concentration by an average of 7% and 2% for tropical and subtropical forests, respectively. Although soil carbon sequestration was higher in the topsoil compared to the subsoil, the relative influence indicated that nitrogen availability strongly impacts the SOC, followed by dissolved organic carbon concentration and mean annual precipitation. This study highlights the critical role of soil N use efficiency processes in promoting soil C accumulation in a forest ecosystem. | Manuscript |
| **INTRODUCTION** | | |  |
| Rationale | 3 | There are many gaps in our knowledge of how N deposition in forests affects soil C sequestration in China.   - The first limitation, they focus more on the region where it has been well reported that there has been a decrease in N deposition (Hole & Engardt 2008) rather than focusing on the hot spot of N sequestration like China, which is the world’s leading N producer and emitter (Liu et al. 2013). - Second recent studies on forest soil C sequestration have overwhelmingly emphasized the impact of chronic N addition on C gain in boreal forests |  |
| Objectives | 4 | The specific objectives of this study are to (1) figure out how C sequestration responds to N addition with forest type, N fertilization rate, and duration; (2) to investigate the relationship between soil C sequestration and controlling factors, as well as their relative importance under N enrichment; and (3) determine how climate factors influence soil C sequestration patterns. |  |
| **METHODS** | | |  |
| Eligibility criteria | 5 | (1) the experiments must be conducted in the field in China, and only field studies performed in forest ecosystems were used; (2) the experiments were performed on forest areas older than 10 years at the start of the experiment; (3) the experiments included a control treatment, and if many levels of N addition were applied in the same experiment, then the measurements for different N application rates were considered as independent observations and (4) the levels and types of N addition and experiment duration were reported or extracted through the figures or tables (Fig. S1). | Manuscript |
| Information sources | 6 | Websites (google, google scholar, and researchgate) (1900–2020) |  |
| Search strategy | 7 | Using the following keywords: “forest” and “carbon” and “nitrogen addition or nitrogen enrichment or nitrogen deposition or nitrogen fertilization or boreal forests, subtropical, temperate, and tropical.” |  |
| Selection process | 8 | Identification, Screening and Included |  |
| Data collection process | 9 | We used Graph Grabber to collect data from graph and figures. This search returned 2086 papers, the titles of which were scanned to eliminate irrelevant papers. |  |
| Data items | 10a | The compiled database of belowground contained 10 variables including with (a) bulk density (BD), (b) total nitrogen (TN), (c) dissolved organic carbon (DOC), (d) soil pH, (e) clay content), (f) sand content, (g), silt content, (h) MWD, SOC and (i) NUE under N enrichment |  |
|  | 10b | All the variables were compared among different forest types (i.e., temperate, and subtropical), N addition forms (NH4NO3 and Urea), N addition rates (<30, 30–70, and >70 kg N ha-1 yr-1), N deposition rate (i.e., <10, 10–20 and >20 kg N ha-1 yr-1) and N fertilization duration (<1, 1–3 and >3 years). Microbial respiration and root respiration variables were excluded from the analysis due to insufficient or lack of data. |  |
| Study risk of bias assessment | 11 | Uncertainties might remain in our study when determining the carbon sequestration rate due to the lack of information on the fraction of N retention in Chinese forest ecosystems. | Manuscript |
| Effect measures | 12 | Specify for each outcome the effect measure(s) (e.g. risk ratio, mean difference) used in the synthesis or presentation of results. |  |
| Synthesis methods | 13a | We searched the peer-reviewed journal articles that studied N addition effects on belowground C dynamics (Table S1) in Web of Science and Google Scholar (1900–2020), using the following keywords: “forest” and “carbon” and “nitrogen addition or nitrogen enrichment or nitrogen deposition or nitrogen fertilization or boreal forests, subtropical, temperate, and tropical.” This search returned 2086 papers, the titles of which were scanned to eliminate irrelevant papers.  The following criteria were used to select appropriate studies and avoid bias in publication: (1) the experiments must be conducted in the field in China, and only field studies performed in forest ecosystems were used; (2) the experiments were performed on forest areas older than 10 years at the start of the experiment; (3) the experiments included a control treatment, and if many levels of N addition were applied in the same experiment, then the measurements for different N application rates were considered as independent observations and (4) the levels and types of N addition and experiment duration were reported or extracted through the figures or tables (Fig. S1). The following framework was used in this study to select, classify the studies, describe, and analyze the data by using the method described by Arksey and O'Malley (2005). This led to a compilation of 1203 field observations from 61 studies (publications) across China. Two types of forests were included in this analysis: temperate, and subtropical (Fig. S1). The means, standard deviations (SD) or standard errors (SE), and sample sizes were reported, calculated, or extracted using OriginPro version 2021 software (Origin Lab Corporation, Northampton, MA, USA) or Graph Grabber version 2.0.2, Quintessa Ltd if the data were graphically presented. |  |
|  | 13b |  |  |
|  | 13c |  |  |
|  | 13d |  |  |
|  | 13e |  |  |
|  | 13f |  |  |
| Reporting bias assessment | 14 |  |  |
| Certainty assessment | 15 |  |  |
| **RESULTS** | | |  |
| Study selection | 16a | Our findings reveal that N inputs enhanced net soil C sequestration by 5.52 to 18.46 kg C kg-1 N, with greater impacts in temperate forests (8.37 to 13.68 kg C kg-1 N), the use of NH4NO3 fertilizer (7.78 kg C kg-1 N), at low N levels (<30 kg C kg-1 N; 9.14 kg C kg-1 N), and in a short period (<3 years; 12.95 kg C kg-1 N). The NUE varied between 0.24 and 13.3 (kg C kg-1 N) depending on the forest type and was significantly controlled by rainfall, fertilizer, and carbon-nitrogen ratio rates. Besides, N enrichment increased SOC concentration by an average of 7% and 2% for tropical and subtropical forests, respectively. Although soil carbon sequestration was higher in the topsoil compared to the subsoil, the relative influence indicated that nitrogen availability strongly impacts the SOC, followed by dissolved organic carbon concentration and mean annual precipitation. Overall, 1203 papers were obtained for this study, but only 61 were retained following the screening stage to exclude irrelevant publications (Fig. 1). | Manuscript |
|  | 16b | Overall, 1203 papers were obtained for this study, but only 61 were retained following the screening stage to exclude irrelevant publications (Fig. 1). | Manuscript |
| Study characteristics | 17 | Cite each included study and present its characteristics.   \| **N°** \| **Citation** \| **N dep** \| **Unit** \| **N form** \| **Forest type** \| **MAT** \| **MAP** \| **Location** \| \| --- \| --- \| --- \| --- \| --- \| --- \| --- \| --- \| --- \| \| 1 \| ([Deng et al., 2010](#_ENREF_7)) \| 29.5 \| kg N ha^-1^ yr^-1^ \| NH_4_NO_3_ \| Subtropical \| 21.5 \| 1750 \| 23°20'N, 113°30'E \| \| 2 \| ([Fu et al., 2020](#_ENREF_12)) \| 18.5 \| kg N ha^-1^ yr^-1^ \| NH_4_NO_3_ \| Subtropical \| 15.7 \| 995.4 \| 31°48'-31°58N, 117°11-117°22'E \| \| 3 \| ([Shi et al., 2019](#_ENREF_31)) \| 8.5 \| kg N ha^-1^ yr^-1^ \| NH_4_NO_3_ \| Boreal \| -5.4 \| 550 \| 51°00′N, 122°03′E \| \| 4 \| ([Mo et al., 2007](#_ENREF_28)) \| 37 \| kg N ha^-1^ yr^-1^ \| NH_4_NO_3_ \| Tropical \|  \| 1927 \| 112°100'E, 23°100'N \| \| 5 \| ([Yan et al., 2016](#_ENREF_48)) \| 13.33 \| kg N ha^-1^ yr^-1^ \| NH_4_NO_3_ \| Temperate \| 12.3 \| 728 \| 48°02′–48°12′ N, 128°58′–129°15′ E \| \| 6 \| ([Liu et al., 2017](#_ENREF_20)) \| 36.3 \| kg N ha^-1^ yr^-1^ \| NH_4_NO_3_ \| Subtropical \| 19.1 \| 1750 \| 26°19′55′′ N, 117° 36′53′′ E \| \| 7 \| ([Gao et al., 2014](#_ENREF_13)) \| 22.6 \| kg N ha^-1^ yr^-1^ \| NH_4_NO_3_ \| Subtropical \|  \|  \| 29°52'N, 121°39'E \| \| 8 \| ([Song et al., 2017](#_ENREF_33)) \| 12.93 \| kg N ha^-1^ yr^-1^ \| NH_4_NO_3_ \| Temperate \| −0.3 \| 805 \| 47° 10′ 50″ N, 128° 53′ 20″ E \| \| 9 \| ([Tu et al., 2011](#_ENREF_37)) \| 25.5 \| kg N ha^-1^ yr^-1^ \| NH_4_NO_3_ \| Subtropical \|  \|  \| 29°95′ N, 103°38′ E \| \| 10 \| ([Lu et al., 2019](#_ENREF_25)) \| 34 \| kg N ha^-1^ yr^-1^ \| NH_4_NO_3_ \| Subtropical \| 26.4 \| 1900 \| 24°22'~24°31'N, 113°05'~113°31'E \| \| 11 \| ([Yu et al., 2020](#_ENREF_49)) \| 40 \| kg N ha^-1^ yr^-1^ \| NH_4_NO_3_ \| Subtropical \| 1700 \| 21.4 \| 23°09′-23°12′N, 112°31′-112°34′E \| \| 12 \| ([Li et al., 2019b](#_ENREF_16)) \| 19 \| kg N hm^-2^ a^-1^ \| NH_4_NO_3_ \| Temperate \| 645.3 \| 4.7 \| 43°41′30″N-43°49′N, 125°26′15″E-125°33′45″E \| \| 13 \| ([Ma et al., 2021](#_ENREF_27)) \| 9 \| kg N ha^-1^ yr^-1^ \| NH_4_NO_3_ \| Tropical \| 19.7 \| 2198 \| 18°43'N, 108°53'E \| \|  \| / \| 16 \| kg N ha^-1^ yr^-1^ \| NH_4_NO_3_ \| Subtropical \| 19.7 \| 2198 \| 27°39'N, 117°57'E \| \|  \| / \| 15 \| kg N ha^-1^ yr^-1^ \| NH_4_NO_3_ \| Temperate \| 19.7 \| 2198 \| 39°58'N, 115°26'E \| \|  \| / \| 7 \| kg N ha^-1^ yr^-1^ \| NH_4_NO_3_ \| Boreal \| 19.7 \| 2198 \| 50°56'N, 121°30'E \| \| 14 \| ([Peng et al., 2017](#_ENREF_30)) \| 10 \| kg N ha^-1^ yr^-1^ \| NH_4_NO_3_ \| Subtropical \| 10 \| 1395 \| 29°32′35″N, 103°15′41″E \| \| 15 \| ([Ma et al., 2018](#_ENREF_26)) \| 14.98 \| kg N ha^-1^ yr^-1^ \| NH_4_NO_3_ \| Subtropical \| 8.7 \| 625 \| 36°40N–36°47′N, 111°59′E–112°05′E \| \| 16 \| ([Li et al., 2019a](#_ENREF_15)) \| 22.6 \| kg N ha^-1^ yr^-1^ \| Urea \| Subtropical \| 0.13 \| 414.98 \| 37°11′N, 102°46′E \| \| 17 \| ([Zhang et al., 2017](#_ENREF_53)) \| 33.5 \| kg N ha^-1^ yr^-1^ \| NH_4_NO_3_ \| Subtropical \| 15.6 \| 1420 \| 30°01′N, 117°21′E \| \| 18 \| ([Lu et al., 2012](#_ENREF_23)) \| 29.5 \| kg N ha^-1^ yr^-1^ \| NH_4_NO_3_ \| Subtropical \| 21 \| 1927 \| 30°01′N, 117°21′E \| \| 19 \| ([Chen et al., 2012](#_ENREF_3)) \| 14.98 \| kg N ha^-1^ yr^-1^ \| NH_4_NO_3_ \| Subtropical \| 21.5 \| 1956 \| 23°10′N, 112°10′E \| \| 20 \| ([Liu et al., 2020](#_ENREF_18)) \| 25 \| kg N ha^-1^ yr^-1^ \| NH_4_NO_3_ \| Boreal \| -2.4 \| 500 \| 51°05′-51°39′ N, 125°07′-125°50′ E \| \| 21 \| ([Chen et al., 2017](#_ENREF_4)) \| 23 \| kg N ha^-1^ yr^-1^ \| NH_4_NO_3_ \| Temperate \| 2.7 \| 871.6 \| 42.517 N, 127.783 E \| \| 22 \| ([Chen et al., 2018](#_ENREF_1)) \| 39 \| kg N ha^-1^ yr^-1^ \| NH_4_NO_3_ \| Subtropical \| 6.6 \| 1490 \| 29'42°N, 103'14°E \| \| 23 \| ([Chen et al., 2019](#_ENREF_5)) \| 23 \| kg N ha^-1^ yr^-1^ \| NH_4_NO_3_ \| Temperate \| 3.6 \| 740 \| 41°42'N, 127°38'E \| \| 24 \| ([Chen et al., 2020](#_ENREF_2)) \| 21.95 \| kg N ha^-1^ yr^-1^ \| NH_4_NO_3_ \| Subtropical \| 17.5 \| 1900 \| 27°42'N, 117°45'E \| \| 25 \| ([Cheng et al., 2018](#_ENREF_6)) \| 20 \| kg N ha^-1^ yr^-1^ \| Urea \| Temperate \| 3.6 \| 700 \| 41°42'N, 127°38'E \| \| 26 \| ([Wang et al., 2016](#_ENREF_41)) \| / \| / \| Urea \| Temperate \| 9.9 \| 548 \| 36°18′N, 111°45′E \| \| 27 \| ([Wang et al., 2019](#_ENREF_40)) \| 117 10 \| kg N ha^-1^ yr^-1^ \| NH_4_NO_3_ \| Subtropical \| 16 \| 1626 \| 30°01'47” N, 117°21'23” \| \| 28 \| ([Du et al., 2014](#_ENREF_8)) \| 20 \| kg N ha^-1^ yr^-1^ \| Urea \| Temperate \| −1.4 \| 450.1 \| 42°10′–42°50′N, 117°12′–117°30′E \| \| 29 \| ([Lu et al., 2010](#_ENREF_24)) \| 31 \| kg N ha^-1^ yr^-1^ \| NH_4_NO_3_ \| Tropical \| 21 \| 1927 \| 23°10'N, 112°10'E \| \| 30 \| ([Wang et al., 2009](#_ENREF_39)) \| 25 \| kg N hm^-2^ a^-1^ \| NH_4_NO_3_ \| Subtropical \| 20.9 \| 1927 \| 23°08′N, 112°35′E \| \| 31 \| ([Fang et al., 2007](#_ENREF_11)) \| 35.6 \| kg N ha^-1^ yr^-1^ \| NH_4_NO_3_ \| Subtropical \| 21 \| 1927 \| 23°19′N, 112°19′E \| \| 32 \| ([Duan et al., 2019](#_ENREF_9)) \| 12.82 \| kg N ha^-1^ yr^-1^ \| NH_4_NO_3_ \| Tropical \| 15.6 \| 1420 \| 22°06'N, 106°43'E \| \| 33 \| ([Zhang et al., 2019a](#_ENREF_52)) \| 7.91 \| kg N ha^-1^ yr^-1^ \| NH_4_NO_3_ \| Temperate \| 11 \| 600 \| 36°040 N, 112°060 E \| \| 34 \| ([Zhou et al., 2019](#_ENREF_56)) \| 40 \| kg N ha^-1^ yr^-1^ \| NH_4_NO_3_ \| Subtropical \| 18.7 \| 2025 \| 26°110'N, 117°280'E \| \| 35 \| ([Fang et al., 2007](#_ENREF_11)) \| 35.6 \| g N m^-2^ yr^-1^ \| NH_4_NO_3_ \| Tropical \| 21 \| 1,927 \| 23°19′N, 112°19′E \| \| 36 \| ([Xiao et al., 2020](#_ENREF_45)) \| 43 \| kg N ha^-1^ yr^-1^ \| NH_4_NO_3_ \| Subtropical \| 16.7 \| 1469 \| 29°16'–29°17'N, 115°42'–15°43'E \| \| 37 \| ([Lu et al., 2013](#_ENREF_21)) \| 33 \| kg N ha^-1^ yr^-1^ \| NH_4_NO_3_ \| Subtropical \| 21 \| 1927 \| 23°10'N, 112°10'E \| \| 38 \| ([Zhu et al., 2021](#_ENREF_58)) \| 5.33 \| kg N ha^-1^ a^-1^ \| Urea \| Temperate \| 2 \| 500 \| 43.47°N, 87.18°E \| \| 39 \| ([Liang et al., 2019](#_ENREF_17)) \| 2 \| g N m^-2^ yr^-1^ \| NH_4_NO_3_ \| Subtropical \| 15.2 \| 1120 \| 36° 52′N, 114° 05′E \| \| 40 \| ([Wang et al., 2017](#_ENREF_43)) \| 12.82 \| kg N ha^-1^ yr^-1^ \| NH_4_NO_3_ \| Subtropical \| 16.5 \| 1200 \| 26◦40 N, 109◦26 E \| \| 41 \| ([Wende et al., 2020](#_ENREF_44)) \| 39.2 \| kg N ha^-1^ yr^-1^ \| NH_4_NO_3_ \| Subtropical \| 17.2 \| 1422 \| 28°06′07''N, 113°02'01''E \| \| 42 \| ([WANG et al., 2015](#_ENREF_42)) \| 12.82 \| kg N ha^-1^ yr^-1^ \| NH_4_NO_3_ \| Subtropical \| 16.5 \| 1200 \| 26°40'–27°09'N, 109°26'–110°08'E \| \| 43 \| ([Zeng et al., 2018](#_ENREF_51)) \| 7 \| kg N ha^-1^ yr^-1^ \| Urea \| Temperate \| -1.4 \| 450.1 \| 42°10′–42°50′N, 117°12′–117°30′E \| \| 44 \| ([Sun et al., 2014](#_ENREF_34)) \| 32 \| kg N ha^-1^ yr^-1^ \| NH_4_NO_3_ \| Tropical \| -1.4 \| 450 \| 42°25'N, 117°15'E \| \| 45 \| ([Mo et al., 2008](#_ENREF_29)) \| 32 \| kg N ha^-1^ yr^-1^ \| NH_4_NO_3_ \| Tropical \|  \| 1927 \| 23°10'N, 112°10'E \| \| 46 \| ([Zhao et al., 2018](#_ENREF_55)) \| 21.2 \| kg N ha^-1^ yr^-1^ \| Urea \| Temperate \| 9.9 \| 662 \| 36°31’N-36°43’ N, 112°01′E-112°15′E \| \| 47 \| ([Lu et al., 2013](#_ENREF_21)) \| 33 \| kg N ha^-1^ yr^-1^ \| NH_4_NO_3_ \| Tropical \| 21 \|  \| 23°10'N, 112°10'E \| \| 48 \| ([Yan et al., 2017](#_ENREF_47)) \| 2.5 \| g N m^-2^ yr^-1^ \| NH_4_NO_3_ \| Temperate \| 13.6 \| 489 \| 51°05'–51°39'N, 125°07'–125°50'E \| \| 49 \| ([Zhang et al., 2019b](#_ENREF_54)) \| 18 \| kg N ha^-1^ yr^-1^ \| NH_4_NO_3_ \| Temperate \|  \|  \| 23°9′41″N, 112°32′36″E \| \| 50 \| ([Tian et al., 2017](#_ENREF_35)) \| 5.5 \| kg N ha^-1^ yr^-1^ \| NH4NO3 \|  \| -5.4 \| 481 \| 50°56′N, 121°30′E \| \|  \| / \| 7 \| kg N ha^-1^ yr^-1^ \| NH4NO3 \|  \| -0.5 \| 654 \| 48°07′N, 129°11′E \| \|  \| / \| 10.6 \| kg N ha^-1^ yr^-1^ \| NH4NO3 \|  \| 9.2 \| 1650 \| 30°01′N, 117°21′E \| \|  \| / \| 16 \| kg N ha^-1^ yr^-1^ \| NH4NO3 \|  \| 18 \| 1889 \| 27°39′N, 117°57′E \| \| 51 \| ([Zhou et al., 2018](#_ENREF_57)) \| 13 \| kg N ha^-1^ yr^-1^ \| NH_4_NO_3_ \| Subtropical \| 16.2 \| 1700 \| 30˚03’N, 102˚59’E \| \| 52 \| ([Fan et al., 2014](#_ENREF_10)) \| 4.2 \| g N m^-2^ yr^-1^ \| Urea \| Subtropical \|  \|  \| 26°30′N, 117°43′E \| \| 53 \| ([Lu et al., 2021](#_ENREF_22)) \| 34.4 \| kg N ha^-1^ yr^-1^ \| NH_4_NO_3_ \| Tropical \| 21.9 \| 1748 \| 23°10'N, 112°10'E \| \| 54 \| ([Zhu et al., 2016](#_ENREF_59)) \| 43.1 \| kg N ha^-1^ yr^-1^ \| NH_4_NO_3_ \| Tropical \| 22.5 \| 1543 \| 22°34'N, 112°50'E \| \| 55 \| ([Yan et al., 2018](#_ENREF_46)) \| 2.5 \| g N m^-2^ yr^-1^ \| NH_4_NO_3_ \| Boreal \| −2.4 \| 489.2 \| 51°05′–51°39′N, 125°07′–125°50′E \| \| 56 \| ([Zeng and Wang, 2015](#_ENREF_50)) \| 1.3 \| g N m^-2^ yr^-1^ \| Urea \| Temperate \| 1.4 \| 450.1 \| 42°10'–42°50'N, 117°12'–117°30'E \| \| 57 \| ([Wang et al., 2018](#_ENREF_38)) \| 32 \| kg N ha^-1^ yr^-1^ \| NH_4_NO_3_ \| Tropical \| 21 \| 1927 \| 23°10′N, 112°10′E \| \| 58 \| ([Liu et al., 2013](#_ENREF_19)) \| 33 \| kg N ha^-1^ yr^-1^ \| NH_4_NO_3_ \| Tropical \| 21 \|  \| 23°109'N, 112°10'E \| \| 59 \| ([Tian et al., 2019](#_ENREF_36)) \| 73 \| kg N ha^-1^ yr^-1^ \| NH_4_NO_3_ \| Tropical \| 21 \| 1930 \| 23°10′N, 112°10′E \| \| 60 \| ([Song et al., 2020](#_ENREF_32)) \| 13 \| kg N ha^-1^ yr^-1^ \| Urea \| Temperate \| − 1.4 \| 450 \| 42° 24.723′ N, 117° 14.844′ E \| \| 61 \| ([Jia et al., 2010](#_ENREF_14)) \| 14.3 \| kg N ha^-1^ yr^-1^ \| NH_4_NO_3_ \| Temperate \| 2.8 \| 723 \| 45°21′-45°25′ N, 127°30′-127°34′E \| \|  \|  \|  \|  \|  \|  \|  \|  \|  \| | Supplementary file |
| Risk of bias in studies | 18 | Standard deviation | Manuscript |
| Results of individual studies | 19 | Soil carbon sequestration varied significantly across the Chinese forests, with different levels of N form, N addition, NUE, and N application time ranging from 6.8 to 11.2 kg C kg-1 N. Soil C sequestration response was significantly higher in temperate forests (10.6: 8.37–13.68 kg C kg-1 N) compared to subtropical forests (5.87: 4.83–7.16 kg C kg-1 N). In addition, C sequestration was more sensitive to NH4NO3, low N addition rate (<30 kg C ha-1 yr-1), high NUE rate (>7 kg C ha-1 yr-1), and short-term N application (<1 year). The relative influence showed that nitrogen availability has the strongest impact on the SOC, followed by DOC> MAP> MAT> clay content> pH> sand content> silt content. |  |
| Results of syntheses | 20a |  |  |
|  | 20b |  |  |
|  | 20c |  |  |
|  | 20d |  |  |
| **DISCUSSION** | | |  |
| Discussion | 23a | The most promising strategy for carbon sequestration in N-limited forest soils is the incorporation of organic inputs in alongside with N deposition. | Manuscript |
|  | 23b | Uncertainties might remain in our study when determining the carbon sequestration rate due to the lack of information on the fraction of N retention in Chinese forest ecosystems. Indeed, no study to date has determined this parameter, which led us to use the values obtained in Europe (50% and 15% for temperate and subtropical forests, respectively) (de Vries et al. 2014; De Vries et al. 2007). It is well known that biological processes control N retention in forest ecosystems, but these processes vary from one site to another. Therefore, although its values were determined in the same forest types, several factors (such as the nitrogen deposition rate, tree species types, climate conditions, vegetation, and microbial uptake) due to the specific sites conditions and environment could influence N retention rate. Moreover, heterotrophic demand for N, soil properties, nonbiological retention of N, land-use change, or plant community can also significantly impact N retention, for instance (Barrett & Burke 2002; de Vries & Bardgett 2016; Johnson et al. 2000; Johnson 1992; Silver et al. 2005; Templer et al. 2008). |  |
|  | 23c |  |  |
|  | 23d |  |  |
| **OTHER INFORMATION** | | |  |
| Support | 24 | National Natural Science Foundation of China (No. 41671295), National Key R. & D Program of China (No. 2017YFD0200106), 111 Project (No. B12007). | Manuscript |
| Competing interests | 25 | Authors confirm no conflict of interest. |  |
| Availability of data, code and other materials | 26 | Upon request |  |

*From:*  Page MJ, McKenzie JE, Bossuyt PM, Boutron I, Hoffmann TC, Mulrow CD, et al. The PRISMA 2020 statement: an updated guideline for reporting systematic reviews. BMJ 2021;372:n71. doi: 10.1136/bmj.n71

For more information, visit: <http://www.prisma-statement.org/>
